# A Power-Optimized Cooperative MAC Protocol for Lifetime Extension in Wireless Sensor Networks

**DOI:** 10.3390/s16101630

**Published:** 2016-10-01

**Authors:** Kai Liu, Shan Wu, Bo Huang, Feng Liu, Zhen Xu

**Affiliations:** 1 School of Electronics and Information Engineering, Beihang University, Beijing 100191, China; yufish2@163.com (S.W.); huangbo_7113@163.com (B.H.); liuf@buaa.edu.cn (F.L.); 2 Beijing Key Laboratory for Network-Based Cooperative Air Traffic Management, Beijing 100191, China; 3 Collaborative Innovation Center of Geospatial Technology, Wuhan 430079, China

**Keywords:** wireless sensor networks, medium access control (MAC), cooperative MAC protocol, transmission power optimization, cooperative node selection, network lifetime, energy efficiency

## Abstract

In wireless sensor networks, in order to satisfy the requirement of long working time of energy-limited nodes, we need to design an energy-efficient and lifetime-extended medium access control (MAC) protocol. In this paper, a node cooperation mechanism that one or multiple nodes with higher channel gain and sufficient residual energy help a sender relay its data packets to its recipient is employed to achieve this objective. We first propose a transmission power optimization algorithm to prolong network lifetime by optimizing the transmission powers of the sender and its cooperative nodes to maximize their minimum residual energy after their data packet transmissions. Based on it, we propose a corresponding power-optimized cooperative MAC protocol. A cooperative node contention mechanism is designed to ensure that the sender can effectively select a group of cooperative nodes with the lowest energy consumption and the best channel quality for cooperative transmissions, thus further improving the energy efficiency. Simulation results show that compared to typical MAC protocol with direct transmissions and energy-efficient cooperative MAC protocol, the proposed cooperative MAC protocol can efficiently improve the energy efficiency and extend the network lifetime.

## 1. Introduction

Wireless sensor networks (WSNs) are widely used in many applications, such as environmental monitoring, traffic control, product quality monitoring, mineral survey and disaster relief [1]. One of its typical features is that each node can continuously work for a few months or up to a few years and its battery cannot be replaced after the exhaustion of battery energy. Therefore, how to prolong network lifetime in the case of constrained power and limited energy is one of key issues in WSNs. Most energy consumption of nodes in WSNs is in the process of communication, i.e., mainly in the aspects of multiple access for nodes to access and use channel, and their corresponding packet transmissions [2]. Therefore, the design of energy-efficient medium access control (MAC) protocols has become a research hotspot in WSNs [3,4,5].

To reduce the energy consumption and improve energy efficiency on multiple access, most MAC protocols use duty cycle mechanism [3]. Duty cycle can be adjusted to adapt to variable traffic load. In the variable load adaptive MAC (VLA-MAC) protocol [4], each node can estimate its traffic load through previous packet inter-arrival time and adaptively adjust its duty cycle. At high traffic load, each sender can transmit multiple data packets to its recipient without sleeping after the successful exchange of request-to-send (RTS) and clear-to-send (CTS) packets. Other nodes go to sleep period after sensing the control packets or data packets. This mechanism reduces delay and unnecessary overhead on overhearing and reservation. At low traffic load, the sender can reserve channel in sync phase, and after successful reservation other unrelated nodes can go to sleep period early, which can adapt to dynamic traffic load and improve energy efficiency. To reduce energy consumption on packet transmissions, in the rate adaptive MAC (RA-MAC) protocol [5], each node estimates the channel gain and successful transmission probability of data packets by overhearing other nodes’ packet transmissions, and sends data packets using the data rate with the minimum energy consumption, thus improving network energy efficiency. Meanwhile, in order to adapt to channel state and improve energy efficiency, the protocol reduces the data rate after a data packet transmission failure and increases the data rate after successful data packet transmission for a certain times. To achieve good energy efficiency and obtain low delivery latency for both normal and emergency packets, a TDMA-based quasi-sleep-preempt-supported (QS-PS) MAC protocol is proposed [6]. In the protocol, a tree structure is built for slot allocation in the starting phase. All nodes transmit their packets in the allocated slots, while entering the Q-Sleep mode in the other slots, which can reduce the clock rate of circuit for preserving some detecting ability. The node with emergency packets can broadcast a special designed m-sequence-based awakening message to wake up the whole network and preempts the right to use the current slot to transmit the packets without interference from other nodes by using a slot preemption strategy. This process continues until the emergency packets reach the root, thus fulfilling the fast delivery of the emergency packets.

In recent years, both multi-input multi-output (MIMO) and cooperative communication techniques can efficiently improve energy efficiency at a certain throughput and bit error rate (BER), by taking advantage of the broadcasting nature of wireless communication and independent fading characteristic of wireless channel to obtain spatial diversity. The MIMO technique employs multiple transmitting and receiving antennas to achieve spatial diversity and thus improves the system capacity and reduces energy consumption. However, in WSNs, due to the limitation of size and cost, it is difficult to install multiple antennas on each node. Therefore, it cannot be widely used in WSNs. In this case, the cooperative communication technique can transform the direct transmission link to the cooperative transmission link with better channel quality by exploiting cooperative nodes to help the sender forward data packets to its recipient, which constructs a virtual MIMO system and reduces energy consumption of data packet transmissions. Moreover, it can optimize local nodes’ transmission power and energy consumption as a whole and reasonably allocate transmission energy consumption among the sender and its cooperative node(s), thereby balancing local nodes’ energy consumption. Therefore, the cooperative communication technique can be used in WSNs to improve energy efficiency and extend network lifetime.

Early research work on cooperative communication techniques primarily focuses on the physical (PHY) layer [7,8]. In order to improve system capacity, decrease BER or reduce energy consumption, cooperative nodes can relay the data packet from the sender to its recipient through amplify-and-forward or decode-and-forward schemes [7], or collaborate with the sender to transmit the data packets to the recipient with the help of distributed space-time coding (DSTC) scheme [9]. Recently, more and more research work has applied cooperative communication techniques to high layers, such as MAC layer [10,11,12,13] and network layer [1,13,14], thereby transforming the diversity gain obtained by the PHY layer to the higher layers. The MAC layer is close to PHY layer and its related protocol is adopted to coordinate multiple nodes sharing the channel, exchanging information and realizing cooperative transmissions, and thus avoiding packet collisions, improving throughput and energy efficiency and reducing delay [15]. Therefore, the design of cooperative MAC protocols has significant influence on the effect of transforming diversity gain to the higher layers. Cooperative MAC protocols for wireless local area networks and mobile ad hoc networks are well studied. However, only a few cooperative MAC protocols are designed for WSNs to improve energy efficiency and extend network lifetime.

Motivated by this, we proposed a power-optimized cooperative MAC (PO-CMAC) protocol for WSNs in this paper. The main work includes two aspects. First, to prolong the lifetime of the WSNs with limited energy, we propose the lifetime-extended transmission power optimization (LETPO) algorithm based on the greedy algorithm and linear programming algorithm. This algorithm balances the energy consumption of the local network by maximizing the minimum residual energy of all the nodes participating in each data packet transmission. Second, based on the algorithm, we propose the PO-CMAC protocol resolving the problem that coordinates multiple nodes to access and use the wireless channel. With the help of a proposed cooperative node selection mechanism, the protocol can choose the nodes with the best channel quality to take part in the cooperative transmissions, thereby reducing the energy consumption of data packet transmissions and improving the nodes’ energy efficiency.

The main contributions of this paper are summarized as follows:

- We consider both energy efficiency (i.e., the average consumed energy during each data packet transmission and the average number of data packets transmitted by each node) and energy utilization (i.e., the ratio of consumed network energy during network lifetime to the total network energy) to extend network lifetime and transmit more data packets in our proposed PO-CMAC protocol. With the help of the proposed LETPO algorithm, the protocol can balance the energy consumption of different nodes and improve the network energy utilization. Additionally, by using the proposed cooperative node selection mechanism, the protocol can select the cooperative nodes with the highest channel gain to participate in each cooperative transmission to reduce the average energy consumption of data packet transmissions and improve the energy efficiency of every node.
- Compared to the single node cooperation mode which is widely used in the current cooperative MAC protocols, we consider the node cooperation mode with multiple optimal cooperative nodes. The number of cooperative nodes taking part in the node cooperation can be changed in this protocol according to the actual optimization results of the proposed LETPO algorithm, thereby taking full advantage of the multiple best cooperative nodes in the network to achieve better effect than the single node cooperation mode.
- We propose a cooperative node selection mechanism to guarantee multiple cooperative nodes to access wireless channel orderly according to their channel gains, which can avoid contention collisions and reduce extra cooperation overhead. According to the contention results, it can also optimize the transmission powers of the cooperative nodes to balance the energy consumption of the local network, which further transfers the cooperative gain and energy utility improved by the LETPO algorithm from the PHY layer to the MAC layer.

The remainder of the paper is organized as follows. In Section 2, we introduce the related work of current cooperative MAC protocols. In Section 3, the network model used in this paper is described. The proposed transmission power optimization algorithm is given in Section 4, and the PO-CMAC protocol is presented in Section 5, followed by performance evaluation in Section 6. Conclusions are summarized in Section 7.

## 2. Related Work

Cooperative MAC protocols tackle two key problems based on MAC protocols, i.e., when to cooperate and how to select the cooperative node(s). To resolve the first problem, it is critical to assess whether the node cooperation could improve the network performance and which kind of network performance is improved. The existing research work mainly focuses on two aspects of network performance, i.e., the network throughput and the transmission reliability [15]. According to different opportunities of selecting cooperation, cooperative MAC protocols can be divided into two categories. The first category decides whether node cooperation is needed during reservation phase of MAC protocols according to whether the node cooperation can obtain cooperative gain. The second category adopts cooperative retransmission after the failure of direct transmission.

In the former cooperative MAC protocols, the sender or the cooperative nodes need to determine whether the cooperative transmission is adopted before the transmission of data packet. If at least one cooperative node can improve throughput or decrease the transmission delay, the cooperative transmission is initiated. Otherwise, the data packet is directly transmitted from the sender to the recipient. Typical protocols include the cooperative MAC (CoopMAC) protocol [16] and the two-relay-based cooperative MAC (2rcMAC) protocol [17]. Furthermore, a packet piggyback mechanism is used in the cooperative MAC-aggregation (CoopMACA) protocol [18] and the active relay-based cooperative MAC (AR-CMAC) protocol [15] to allow the cooperative node to send its own data packet without reservation, thus avoiding reservation collision and further improving throughput. Based on the traditional cooperative node selection criterion (i.e., throughput improvement), the optimal relay selection MAC (ORS-MAC) protocol [19] also takes into account the transmission error probability, thus guaranteeing the reliability while improving the throughput.

The latter cooperative MAC protocols adopt the concept of cooperative automatic repeat request (ARQ). If the direct transmission failed and there existed cooperative nodes to successfully receive the data packets, the cooperative transmission is employed to improve successful transmission probability. Typical protocols are the cooperative diversity MAC (CD-MAC) protocol [20], the differentiated cooperative MAC (DC-MAC) protocol [21], the cooperative MAC protocols used in time division multiple access (TDMA) systems [22,23], the persistent relay carrier sensingmultiple access (PRCSMA) protocol [24] and the network coding-based cooperative ARQ MAC (NCCARQ-MAC) protocol [25]. In CD-MAC protocol, each sender selects its cooperative node by overhearing packet transmissions of its neighbors with respect to link quality, and retransmits the data packet using DSTC with the cooperative node if the direct transmission fails. In the cooperative TDMA protocols [22,23], the idle cooperative node of the sender retransmits the failed data packets in its timeslot, which guarantees the transmission reliability and avoids the throughput reduction. The PRCSMA and NCCARQ-MAC protocols modify the basic rules of the IEEE 802.11 MAC protocol to execute a distributed cooperative ARQ scheme in wireless networks in order to enhance their performance [24,25]. Network coding techniques are employed to benefit the energy efficiency of the NCCARQ-MAC protocol, as the number of the delivered bits increases by combining multiple data packets [25].

According to the method to select cooperative nodes, cooperative MAC protocols can be divided into three categories: the sender-specified method, the recipient-specified method, and the cooperative node contention method. In early cooperative MAC protocols, such as the CoopMAC protocol [16] and the relay-enabled distributed coordination function (rDCF) protocol [26], the sender is responsible for the selection of the cooperative nodes based on a best cooperative node table (i.e., CoopTable) maintained by overhearing the packet transmissions of its neighbor nodes. Before the beginning of data packet transmissions, the sender looks up the CoopTable and applies the preselected best cooperative node to the cooperative transmission. In the recipient-specified cooperative MAC protocols, the recipient is responsible for selecting the best cooperative node for cooperative transmissions, like the on-demand cooperation with a selection of relays initiated by the destination equipment (OXIDE) protocol [27]. In this protocol, each recipient maintains a best cooperative node table based on its overhearing of packet transmissions. If the direct transmission failed, the recipient selects the best cooperative node from the table and informs it to retransmit the data packet. The drawback of using the best cooperative node table is that constructing the table consumes the extra computing and storage resources, and the information of the best cooperative nodes is possibly out of date. In recent cooperative MAC protocols, such as 2rcMAC protocol [17], CoopMACA protocol [18], the rapid cooperation-differentiated MAC (RCD-MAC) protocol [28], the priority-differentiated cooperative MAC protocol with contention resolution (CRP-CMAC) [29] and the cooperative MAC protocol with rapid relay selection (RRS-CMAC) [30], each potential cooperative node contends to participate in the cooperative transmission based on the instantaneous channel information. The better the channel quality is, the more possible it becomes the final cooperative node. This method is easy to operate and the information of the best cooperative node is not out-of-date. To avoid the packet collisions in the process of cooperative node contention and improve the MAC performance of cooperative transmissions, collision resolution schemes [28,29,30] are used in this method.

From the above, we can see that most current cooperative MAC protocols mainly focus on improving the throughput and transmission reliability other than improving the energy efficiency. Considering that saving energy is the main challenge in WSNs, the cooperative MAC protocols for the purpose of improving the energy efficiency and extending the network lifetime are more and more proposed.

The objective of energy-efficient cooperative MAC protocols is twofold: optimize energy consumption for each data packet transmission and optimize the entire network lifetime. The first takes advantage of diversity gain generated by node cooperation to reduce the energy consumption for each data packet transmission by choosing the best cooperative node and thus improves the energy efficiency of entire network at a certain throughput and packet error rate (PER) [31,32,33,34]. Opportunistic energy-efficient (OEE) relay selection cooperative communication scheme [31] defines the energy efficiency of data packet transmissions as the ratio of the channel capacity to the transmission power, and selects the nodes with the highest energy efficiency for cooperative transmissions to achieve the objective of improving the energy efficiency. Yang et al. [32] select two groups of neighboring nodes near the sender and the recipient, respectively, to construct a virtual MIMO system. In this process, there may be several different groups of nodes satisfying the PER requirement. The protocol can select the two cooperative groups with the least energy consumption for cooperative transmissions to improve energy efficiency. The link-utility-based cooperative MAC (LC-MAC) protocol [33] considers both throughput and energy efficiency. It calculates the weighted sum of the achieved throughput and needed energy by cooperating with a certain cooperative node and defines it as the link-utility of this cooperative link. Each candidate calculates the optimal transmission power and data rate for participating in cooperation to make its link-utility the largest. The node with the largest link-utility among all candidates will access channel firstly and take part in cooperation after contention, which results in the optimum throughput and energy efficiency of the entire network. The authors in [34] adopt the model of cooperative retransmission and random network coding. The sender repeatedly transmits its data packet until its recipient or one of its cooperative nodes successfully receives it. If the recipient receives the data packet firstly, the data packet transmission ends. Otherwise, the cooperative node repeatedly transmits the data packet until the recipient successfully receives it. The protocol can obtain the average energy consumption based on the successful transmission probability of data packets among the sender, recipient and cooperative node, and minimize it by optimizing the transmission power of the sender and cooperative node. Thus, it can achieve the objective of energy efficiency improvement. However, in this type of cooperative MAC protocols, residual energy information (REI) of nodes is not considered and part nodes in the network drain out their energy more quickly than the others because of more frequent cooperative transmissions, thus decreasing the network lifetime.

The second category of cooperative MAC protocols takes advantage of cooperation among nodes to balance local network energy consumption, avoids some nodes consuming their energy too quickly and makes full use of each node’s energy in the whole network, thereby improving network energy utilization and extending network lifetime [35,36,37]. Given that the nodes with higher transmission rate consume more energy than those with lower transmission rate due to more frequent cooperative transmissions, the lifetime extended cooperative MAC (LECMAC) protocol [35] reduces the opportunity of a node to participate in cooperation transmissions when its residual energy is low by increasing its cost function to extend the network lifetime. However, this protocol balances the local network energy consumption only when the residual energy is very little. Therefore, its effect to extend the network lifetime is limited. The authors in [36] employ the greedy algorithm to optimize transmission power of the sender and a single cooperative node in order to balance the energy consumption of the local network, improve energy utilization and extend network lifetime. However, this protocol only considers the residual energy of nodes and does not consider the average energy consumption on each data packet transmission. Therefore, it cannot guarantee that the entire network can transmit more data packets in the presence of the same initial energy. That is to say, the protocol does not consider the energy efficiency of data packet transmissions. The delay- and energy-aware cooperative MAC (DEC-MAC) protocol [37] does not adopt transmission power optimization and attempts to balance the energy consumption of the sensor nodes by taking into account a node’s residual energy and packet delivery delay as part of the relay selection metric, thus increasing the network lifetime. Its relay selection algorithm exploits the process of elimination and the complementary cumulative distribution function for determining the most optimal relay within the shortest time period.

Both the wireless sensor network cooperative MAC (WcoopMAC) protocol [38] and the total power control MAC (TPC-MAC) protocol [39] consider both the energy efficiency of each node and the energy utilization of the entire network. In these distributed cooperative MAC protocols, the transmission powers are optimally allocated to source and relay nodes with the objective of minimizing the total transmission power under the average BER constraint. Based on this, both channel state information (CSI) and residual energy information (REI) of sensor nodes are considered to choose one cooperative node for cooperative transmission, which can significantly prolong network lifetime compared with non-cooperative MAC protocols.

In summary, the existing research work on improving energy efficiency and extending network lifetime can be improved in the following aspects. Firstly, the existing cooperative MAC protocols mainly use single cooperative node and do not consider the cooperation of multiple cooperative nodes. By exploiting the potential multiple cooperative nodes, the cooperative transmissions can improve the energy efficiency and extend network lifetime more effectively. Furthermore, with the application of randomized DSTC (RDSTC) technique [40] to cooperative communication, the cooperative MAC protocols using multiple cooperative nodes are feasible [41,42]. Secondly, most of existing cooperative MAC protocols consider either the energy efficiency of each node or the energy utilization of the entire network, and lack a comprehensive consideration of both factors. In general, in order to optimize energy consumption for each data packet transmission, transmission power optimization should be adopted in the cooperative MAC protocols and channel quality (or channel state information) should be considered in the process of relay selection, and in order to optimize the entire network lifetime, the residual energy information should be considered. 

Therefore, this paper proposes a power-optimized cooperative MAC (PO-CMAC) protocol based on multiple cooperative nodes and considering both the energy consumption of each data packet transmission and network energy utilization. Each node located in one-hop transmission range of both the sender and its recipient contends to become a cooperative node and participate in cooperative transmission according to the instantaneous channel quality between itself and the sender/recipient, thus reducing the transmission energy consumption and improving energy efficiency. Meanwhile, the sender can optimize its transmission power and each cooperative node’s transmission power according to the proposed LETPO algorithm, thus balancing the energy consumption of the local nodes. Through the above two points, the PO-CMAC protocol can efficiently extend entire network lifetime.

## 3. Network Model

In WSNs, all nodes share a wireless channel to exchange control packets and data packets. Each node can estimate the channel gain between itself and other nodes based on the signal-to-noise ratio (SNR) of received control packets. For simplicity, we assume that the communication channels between any two nodes are bidirectionally symmetrical, i.e., they have the same channel gain, and the channel is invariant during the transmission session of each data packet. The recipient can decode data packets sent from the sender and its cooperative nodes by maximum ratio combining (MRC) technique.

Each node in WSNs has a half-duplex transceiver with the maximum transmission power *P*_max_. Its transmission power can be continuously adjusted within the range of *P*_max_ according to the actual requirement. All nodes have the same initial energy, and their batteries cannot be charged if exhausted. If the residual battery energy of a node is insufficient to send the current packet, we consider that its battery energy is exhausted. For simplicity, this paper only considers the energy efficiency of WSNs in the transmission of information, and ignores the energy consumed in sensing and data processing.

**Figure 1:**
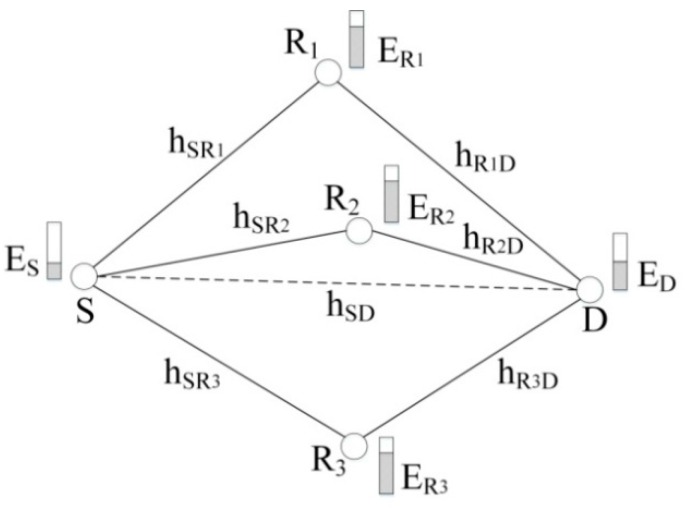
Cooperative transmission model.

Figure 1 shows the cooperative transmission model adopted in this paper. During the current data packet transmission, there are several one-hop neighbor nodes (i.e., R_1_, R_2_ and R_3_) of both the sender S and its recipient D, and the current energy of these nodes are *E*_S_, *E*_D_, $E_{R_{1}}$, $E_{R_{2}}$ and $E_{R_{3}}$, respectively. In traditional direct transmission model, the sender S transmits its data packets to its recipient D directly. The corresponding channel gain is *h*_SD_, and can be obtained by the recipient from receiving cooperative RTS (CRTS) packet and specified in its cooperative CTS (CCTS) packet for the sender and cooperative nodes. In the cooperative transmission model, the sender S transmits its data packets with a lower transmission power than that in the direct transmission model, and the recipient cannot decode the data packets but receive and save the data packets. Some common neighbor nodes of S and D (such as R_1_, R_2_ and R_3_ in Figure 1) can decode the data packets successfully, and forward them to the recipient. The corresponding channel gains are $h_{{\text{SR}}_{i}}$ and $h_{R_{i}D}$ (*i* = 1, 2, 3), respectively. Channel gain *h*_SR_ can be obtained by the cooperative node from receiving CRTS packet and specified in its help-to-send (HTS) packet for the sender and recipient. Channel gain *h*_RD_ is assumed to be equal to channel gain *h*_DR_, which can be obtained by the cooperative node from receiving CCTS packet. For asymmetric wireless channels, the sender and cooperative node can obtain channel gain *h*_RD_ by this way: the recipient obtained channel gain *h*_RD_ from recently received control packets or data packets from the cooperative node and specifies this value in its CCTS packet. The recipient can use the MRC technique to decode the data packets from the sender and these cooperative nodes. This two-hop transmission method is the cooperative transmission model of multiple nodes used in this paper.

In order to guarantee the recipient successfully decoding the data packets, improve the node energy efficiency and extend the network lifetime, the key issues in this cooperative transmission model include how much transmission power the sender should use to transmit data packets, which common neighbor nodes should take part in the cooperative transmission, and how much transmission power each cooperative node should use to forward the data packets. To resolve these issues, we propose the LETPO algorithm and PO-CMAC protocol.

## 4. Lifetime-Extended Transmission Power Optimization Algorithm

### 4.1. Problem Description

In this paper, the network lifetime is defined as the duration from the beginning time of the network operation to the time when the first node exhausts its energy [36,43]. Based on this definition and the above cooperative transmission model, the key issue to extend the network lifetime is to select the cooperative nodes with the best channel quality in order to reduce the energy consumption of each data packet transmission and improve node energy efficiency, and meanwhile, optimize the transmission powers of the sender and the cooperative nodes to guarantee that each node consumes its energy evenly and avoid parts of nodes exhausting their energy while the other nodes still have much residual energy.

Network lifetime is a global variable, which is related to the data packet generation of each node and network traffic load. However, network traffic load distribution is determined by actual application, and each node cannot predict its future data packet arrival rate. In this case, in order to extend network lifetime, we can use the local optimization to replace the global optimization according to the greedy algorithm. That is to say, we should maximize the minimum residual energy among the sender and cooperative nodes during the current data packet transmission [36,43]. This strategy can guarantee that the local energy of network are consumed evenly and avoid exhausting the energy of some nodes too early.

The minimum residual energy *e* of the sender and cooperative nodes after the current data packet transmission can be expressed as:

(1) $$e=\text{min}\{E_{s}-P_{s}T,E_{R_{1}}-P_{R_{1}}T,\cdot \cdot \cdot ,E_{R_{i}}-P_{R_{i}}T,\cdot \cdot \cdot ,E_{R_{N}}-P_{R_{N}}T\},i\in \left[1,N\right]$$

where *E*_S_ is the residual energy of the sender before the current data packet transmission, *P*_S_ is the transmission power of the sender to be used during the current data packet transmission, and *T* is the duration of the data packet transmission. Then, *E*_S_ − *P*_S_*T* represents the residual energy of the sender after the current data packet transmission. Likewise, $E_{R_{i}}$ − $P_{R_{i}}T$ represents the residual energy of the cooperative node R_i_ (*i* = 1, 2, 3, …) after the current cooperative transmission using a transmission power $P_{R_{i}}$. In order to maximize the network lifetime, the proposed transmission power optimization algorithm should optimize the transmission power *P*_S_ and $P_{R_{i}}$ to guarantee the value of *e* to be maximum after the current data packet transmission, i.e., the ultimate objective of the LETPO algorithm is:

(2) $$\underset{\left\{P_{S},P_{R_{1}},P_{R_{2}},\cdots \right\}}{\text{max}}e,\;i\in \left[1,N\right]$$

### 4.2. Constraint Conditions of Transmission Power

The optimal transmission powers *P*_S_ and $P_{R_{i}}$ should satisfy the constraint conditions in two aspects. On one hand, they are large enough to guarantee the SNR of the received signal satisfying a certain BER. On the other hand, they are within the range of the maximum transmission power *P*_max_.

According to Shannon theorem, to ensure cooperative nodes to receive data packets correctly, the transmission power of the sender should satisfy the following constraint condition:

(3) $$R\leq \frac{1}{2}{\text{log}}_{2}\left(1+\frac{P_{S}{\left\|h_{{\text{SR}}_{i}}\right\|}^{2}}{N_{0}}\right),i\in \left[1,N\right]$$

where *R* is the bandwidth utilization between S and D, $h_{{\text{SR}}_{i}}$ is the channel gain between the sender and its cooperative node R_i_, and *N*_0_ is the power of Gaussian white noise. In the cooperative transmission, each data packet transmission is composed of two–hop transmissions, i.e., from the sender to the cooperative node and then from the cooperative node to the recipient, and each one-hop transmission only occupies a half of the channel resource. Therefore, there is a factor 1/2 in Equation (3).

Deduced from Equation (3), the received SNR of a cooperative node should satisfy the following condition:

(4) $$\frac{P_{S}{\left\|h_{{\text{SR}}_{i}}\right\|}^{2}}{N_{0}}\geq 2^{2R}-1,i\in \left[1,N\right]$$

In the cooperative transmission, the recipient receives the signals from the sender and cooperative nodes, and decodes the signals by the MRC technique. Therefore, the transmission powers of the sender and cooperative nodes should satisfy the following condition:

(5) $$R\leq \frac{1}{2}{\text{log}}_{2}\left(1+\frac{P_{S}{\left\|h_{\text{SD}}\right\|}^{2}+\sum_{i}P_{R_{i}}{\left\|h_{R_{i}D}\right\|}^{2}}{N_{0}}\right)$$

That is to say, the received SNR at the recipient should satisfy the condition:

(6) $$\frac{P_{S}{\left\|h_{\text{SD}}\right\|}^{2}+\sum_{i}P_{R_{i}}{\left\|h_{R_{i}D}\right\|}^{2}}{N_{0}}\geq 2^{2R}-1,\;i\in \left[1,N\right]$$

where *h*_SD_ is the channel gain between the sender S and its recipient D, and $h_{R_{i}D}$ is the channel gain between the cooperative node R_i_ and the recipient D.

Meanwhile, all nodes in the network should adjust their transmission powers in range of the maximum transmission power as follows:

(7) $$0<P_{S}\leq P_{\text{max}},$$

(8) $$0\leq P_{R_{i}}\leq P_{\text{max}}.$$

The transmission power of the sender *P*_s_ is always positive because the sender always needs to transmit data packets. However, the transmission power of a cooperative node $P_{R_{i}}$ can be zero. If the transmission power of a cooperative node is zero, this node does not forward the data packet to the recipient in the cooperative transmission.

### 4.3. Constraint Conditions of Channel Gain and Residual Energy of Cooperative Nodes

A cooperative node can take part in the cooperative transmission and then achieve the objective of improving the system capacity and extending the network lifetime only when its residual energy and channel gain between itself and the sender/recipient satisfy certain conditions.

Firstly, in order to extend network lifetime, the cooperative node should have enough residual energy to participate in the cooperative transmission and forward the data packets. That is to say, the residual energy of the cooperative node before the cooperative transmission should be more than that of the sender after transmitting the data packets directly, i.e.,

(9) $$E_{S}-P_{D}T<E_{R_{i}},\;i\in [1,N]$$

where *P*_D_ is the transmission power of the sender in direct transmission, which can be deduced from the Shannon theorem as follows:

(10) $$R\leq {\text{log}}_{2}\left(1+\frac{P_{D}{\left\|h_{\text{SD}}\right\|}^{2}}{N_{0}}\right).$$

Then we get:

(11) $$P_{D}\geq \frac{N_{0}\left(2^{R}-1\right)}{{\left\|h_{\text{SD}}\right\|}^{2}}.$$

Secondly, the cooperative node transforms the packet transmission between the sender and its recipient from one-hop to two-hop. In order to improve the system capacity, the channel gain of the cooperative node between itself and the corresponding sender/recipient should be higher than that of the direct link between the sender and its recipient, i.e.,

(12) $${\left\|h_{{\text{SR}}_{i}}\right\|}^{2}>{\left\|h_{\text{SD}}\right\|}^{2},$$

(13) $${\left\|h_{R_{i}D}\right\|}^{2}>{\left\|h_{\text{SD}}\right\|}^{2}.$$

Moreover, the energy consumption of the cooperative transmission should be constrained. In the cooperative transmission, the energy consumption of the sender should be less than that in the direct transmission, i.e.,

(14) $$P_{S}\times \frac{L}{2RB}<P_{D}\times \frac{L}{RB},$$

where *L* is the length of data packets, and *B* is the bandwidth of the channel.

If a cooperative node R_i_ participates in the cooperative transmission, according to Equation (3), in order to guarantee its correct packet decoding, the transmission power of the sender should satisfy the following condition:

(15) $$P_{S}\geq \frac{N_{0}\left(2^{2R}-1\right)}{{\left\|h_{{\text{SR}}_{i}}\right\|}^{2}}.$$

Substituting Equations (11) and (15) into Equation (14), we have:

(16) $$\frac{{\left\|h_{\text{SD}}\right\|}^{2}}{{\left\|h_{{\text{SR}}_{i}}\right\|}^{2}}<\frac{2(2^{R}-1)}{2^{2R}-1}=\frac{2}{2^{R}+1}.$$

Compared to Equation (12), Equation (15) is a strict constraint. Furthermore, the channel gains of all potential cooperative nodes’ links should satisfy Equations (12), (13) and (16).

Equations (9), (12), (13) and (16) restrict the residual energy and the channel gains of all cooperative nodes’ links. Equations (3)–(8) give the feasible region of the transmission powers of the sender and its multiple cooperative nodes. The optimal values of *P*_S_ and $P_{R_{i}}$ can be calculated in the feasible region to maximize *e*, and thus extend the network lifetime.

## 5. Power-Optimized Cooperative MAC Protocol

The above LETPO algorithm achieves the objective of optimizing the transmission powers of both the sender and its multiple cooperative nodes theoretically. However, during the execution of the algorithm, each node should transmit its channel gain and residual energy to the sender through a control packet. Then the sender performs the transmission power optimization algorithm with these parameters and declares the optimization results to the cooperative nodes with another control packet. Finally, the sender and cooperative nodes transmit the data packets by using their optimized transmission powers, i.e., the sender transmits the data packets using the optimized transmission power *P*_S_ and each cooperative node R_i_ relays the data packets using the optimized transmission power $P_{R_{i}}$.

To perform the LETPO algorithm efficiently, there are still two issues to be addressed. Firstly, if there are many potential cooperative nodes satisfying the constraint condition shown in Equations (9), (12), (13) and (16), how to select parts of them to transmit control packets to the sender, thus avoiding too much delay and energy overhead. Secondly, we should determine the transmission sequence of these control packets to avoid packet collisions.

**Figure 2:**
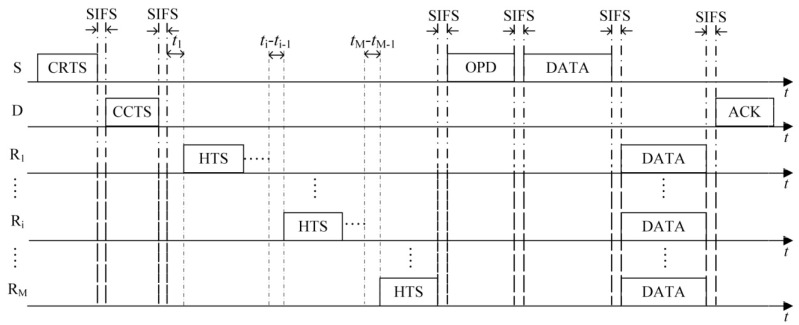
Packet transmissions of power-optimized cooperative MAC (PO-CMAC) protocol.

To address the above issues, execute the LETPO algorithm more efficiently and extend the network lifetime for WSNs, we propose the PO-CMAC protocol. As shown in Figure 2, the protocol includes three phases, namely, the channel reservation phase, the cooperative node selection phase, and the data packet transmission phase.

### 5.1. Channel Reservation Phase

When the sender S has a data packet to send and senses the channel idle, it sends the cooperative RTS (CRTS) packet to its recipient D like IEEE 802.11 distributed coordination function (DCF) protocol. On receiving the CRTS packet correctly, the recipient replies with a cooperative CTS (CCTS) packet if the channel is available from its perspective. With the help of the CRTS/CCTS handshake, other nodes in the network know the data packet transmission between S and D and set their own network allocation vectors (NAVs) to avoid packet transmission collisions. Meanwhile, S and D can estimate the channel gain *h*_SD_ by the received SNR of the CRTS and CCTS. The node R_i_ receiving both CRTS and CCTS knows that it is the common neighbor node of S and D and estimates the channel gain $h_{{\text{SR}}_{i}}$ and $h_{R_{i}D}$. The CRTS packet contains the residual energy of the sender *E*_S_, and the CCTS packet contains the channel gain *h*_SD_. With the information in control packets, the common neighbor nodes R_i_ of S and D can determine whether its corresponding parameters satisfy Equations (9), (12), (13) and (16). If satisfied, the node R_i_ can take part in the process of cooperative node contention.

### 5.2. Cooperative Node Selection Phase

In the cooperative node selection phase, the nodes satisfying the constraint conditions of cooperation contend to be cooperative nodes by sending help-to-send (HTS) packet to the sender. There are two issues to be addressed in this process, i.e., the contention access delay of the HTS packet transmission and the method to determine the optimal group of cooperative nodes.

#### 5.2.1. Contention Access Delay of HTS Packet Transmission

If all the common neighbor nodes R_i_ satisfying the constraint conditions of cooperation send their HTS packets to the sender, there will be too much unnecessary delay, energy overhead and HTS packet collisions. To avoid this case, the maximum number of HTS packets sent to the sender during the cooperative node selection phase is set to be *M* in the PO-CMAC protocol. The value of *M* can be set in advance based on the network scale, the node density and the traffic load distribution of the network.

Moreover, a mechanism is needed to evaluate the ability of all common neighbor nodes to take part in the cooperative transmissions, in order to guarantee the nodes with higher cooperative gain to access the channel and send the HTS packet earlier to participate in the cooperative transmissions, thus extending the network lifetime.

The length of the network lifetime can be reflected by the total number of transmitted data packets *n* during the whole network lifetime. For simplicity, it can be formulated as follows:

(17) $$n=\frac{\eta E_{0}}{E_{\text{PKT}}},$$

where *E*_0_ is the initial energy of the network, *η* is the energy utilization of the network, and *E*_PKT_ is the average energy consumption of each data packet transmission.

Equation (17) shows that, with a certain initial energy, the total number of data packet transmissions is proportional to the energy utilization of the network and inversely proportional to the energy consumption of each data packet transmission. The LETPO algorithm balances the local network energy consumption by maximizing the minimum residual energy of all related nodes according to the greedy algorithm. In this process, the energy utilization is fully considered and all nodes in the network drain out their residual energy as evenly as possible, thus extending the network lifetime. However, it overlooks the energy consumption during each data packet transmission. In other words, the LETPO algorithm concerns the energy utilization more than the energy efficiency. Therefore, in the cooperative node selection phase, we should focus on the energy consumption of each data packet transmission cooperated by the selected cooperative nodes through the corresponding cooperative links. That is to say, we also consider the energy efficiency improvement in the cooperative node selection phase.

The energy consumption of each data packet transmission cooperated by a single cooperative node R_i_ can be expressed as:

(18) $$E_{{\text{PKT-R}}_{i}}=\left(P_{{\text{SR}}_{i}}+P_{R_{i}D}\right)\frac{L}{2RB},$$

where *P*_SRi_ need to satisfy Equation (3), and the transmission power *P*_RiD_ of the cooperative node *R*_i_ needs to satisfy the following condition:

(19) $$R\leq \frac{1}{2}{\text{log}}_{2}\left(1+\frac{P_{{\text{SR}}_{i}}{\left\|h_{\text{SD}}\right\|}^{2}+P_{R_{i}D}{\left\|h_{R_{i}D}\right\|}^{2}}{N_{0}}\right).$$

Considering the constraint conditions of the channel gain *h*_SRi_ and *h*_RiD_ in Equations (12), (13) and (16), to minimize *E*_PKT-Ri_ in Equation (18), *P*_SRi_ and *P*_RiD_ should be set to the following value:

(20) $$P_{{\text{SR}}_{i}}=\frac{\left(2^{2R}-1\right)N_{0}}{{\left\|h_{{\text{SR}}_{i}}\right\|}^{2}},$$

(21) $$P_{R_{i}D}=\left(2^{2R}-1\right)N_{0}\frac{{\left\|h_{{\text{SR}}_{i}}\right\|}^{2}-{\left\|h_{\text{SD}}\right\|}^{2}}{{\left\|h_{R_{i}D}\right\|}^{2}{\left\|h_{{\text{SR}}_{i}}\right\|}^{2}}.$$

Substituting Equations (20) and (21) into Equation (18), we get:

(22) $$E_{{\text{PKT-R}}_{i}}=\left(2^{2R}-1\right)N_{0}\frac{({\left\|h_{{\text{SR}}_{i}}\right\|}^{2}+{\left\|h_{R_{i}D}\right\|}^{2}-{\left\|h_{\text{SD}}\right\|}^{2})L}{2{\left\|h_{R_{i}D}\right\|}^{2}{\left\|h_{{\text{SR}}_{i}}\right\|}^{2}RB}.$$

The above equation shows the minimum energy consumption of one data packet transmission cooperated by single cooperative node R_i_ through its cooperative link is $E_{{\text{PKT-R}}_{i}}$. Its value is related to the channel gain between the cooperative node R_i_ and the corresponding sender/recipient. Namely, $E_{{\text{PKT-R}}_{i}}$ represents the comprehensive channel quality of cooperative link. The smaller it is, the more energy efficientthis cooperative link is, i.e., the higher cooperative gain this cooperative node has to participate in the cooperative transmission, and thus the sooner the cooperative node should transmit its HTS packet to the sender to contend for cooperative transmission.

For this reason, in the cooperative node selection phase, each node needs to wait a certain access delay *t*_Ri_ as follows:

(23) $$t_{R_{i}}=\frac{E_{{\text{PKT-R}}_{i}}}{2P_{\text{max}}\frac{L}{2RB}}T_{W},$$

where *T*_W_ is the maximum waiting time allowed for cooperative nodes to contend to access the channel. Each cooperative node calculates its access delay $t_{R_{i}}$ at the beginning of the contention and start to wait an interval of $t_{R_{i}}$. At the same time, each cooperative node senses the channel. When it senses the HTS packet transmission from other nodes, it stops counting the access delay and continues as soon as the HTS packet transmission ends. When the counting of a node’s access delay ends and the contention phase does not finish, the node transmits its HTS packet to the sender. As shown in Figure 2, if the access delays of all potential cooperative nodes in an order from small to large are *t*_1_, *t*_2_, …, *t*_i_, …, and *t*_M_, the first HTS packet is transmitted at *t*_1_ after the beginning of contention phase, and the second HTS packet is transmitted at *t*_2_ − *t*_1_ after the end of the first HTS packet transmission. In the same way, the *M*-th HTS packet is transmitted at *t*_M_ − *t*_M-1_ after the end of the (*M* − 1)-th HTS packet. This method can avoid the HTS packet collisions from different cooperative nodes.

#### 5.2.2. Optimal Cooperative Node Group

All the cooperative nodes that transmit their HTS packet successfully to the sender from the beginning of the cooperative node contention phase to its end form the optimalcooperative node group to participate in the power-optimized cooperative transmission. According to different ending manners of the cooperative contention phase, there are three cases to determine the optimal cooperative node group.

**Figure 3:**
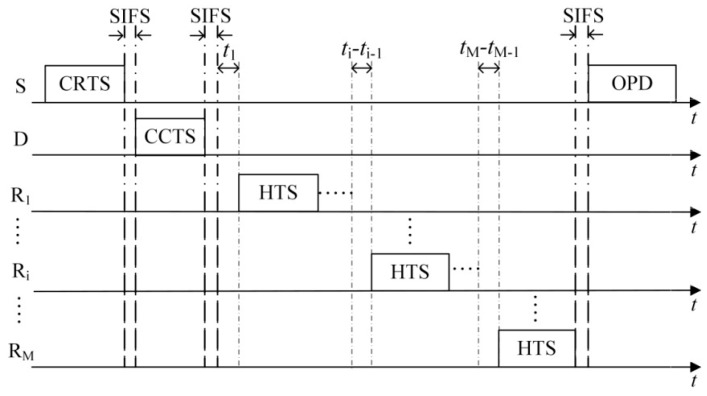
First case of optimal cooperative node group: The number of cooperative nodes reaches *M*.

**Figure 4:**
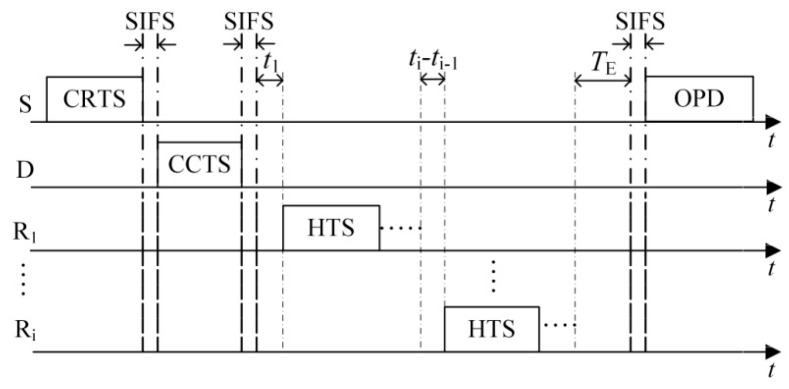
Second case of optimal cooperative node group: No cooperative nodes with higher cooperative gain contend within a time interval of *T*_E_.

**Figure 5:**
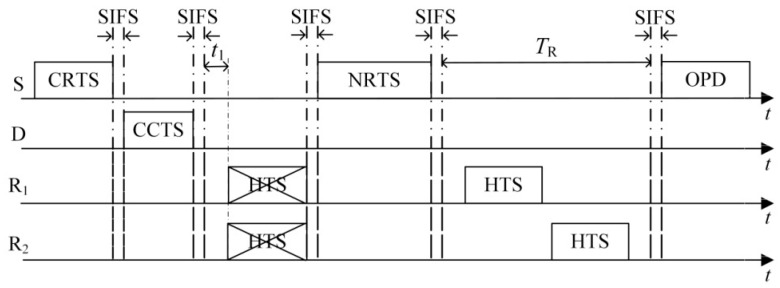
Third case of optimalcooperative node group: help-to-send (HTS) packet transmission collisions occur.

(1) The first case is that the number of HTS packets transmitted to the sender reaches the predefined maximum number of selected cooperative nodes *M*. As shown in Figure 3, on receiving the *M-*th HTS packet, the sender calculates the optimal transmission powers of itself and all the cooperative nodes based on the information of *M* HTS packets (including the corresponding cooperative nodes’ residual energy and channel gain) and the channel gain estimated by itself. Then it broadcasts the optimization results to the cooperative nodes through an optimized-power-declaration (OPD) packet. Meanwhile, all the other cooperative nodes that have not transmitted their HTS packets stop their predefined HTS packet transmissions because they sensed *M* HTS packet transmissions and received the OPD packet.
(2) As shown in Figure 4, the second case lies in that there are no HTS packet transmissions during the time interval of *T*_E_ after a certain HTS packet transmission. When the cooperative nodes that prepare to transmit their HTS packets sense this phenomenon, they will not transmit their HTS packets anymore. Because the sequence of HTS packet transmissions reflects the cooperative gains of the nodes to take part in the cooperative transmission, if the sender has received a few HTS packets, it indicates that some cooperative nodes with higher cooperative gain can participate in the cooperative transmission. In this case, the waiting time for the subsequent HTS packet transmissions should decrease gradually in order to avoid wasting too much time on waiting for the HTS packets of the cooperative nodes with lower cooperative gain and thus leading to more overhead than their incurred cooperative gain. *T*_E_ can be set as follows:
  (24) $$T_{E}=\frac{M-k}{M}\left(T_{W}-t_{k}\right),$$
  where *k* is the number of already-transmitted HTS packets in the network. The value of *T*_E_ decreases as *k* increases, which illustrates that the more HTS packets have been received by the sender, the shorter the time of waiting for the subsequent HTS packet transmissions is. If there are no HTS packet transmissions during an interval of *T*_E_, the cooperative contention phase prematurely ends. Furthermore, based on *k* HTS packets, the sender calculates the optimal transmission powers of itself and its *k* selected cooperative nodes, and declares the results through an OPD packet.
(3) Figure 5 shows the third case that the HTS packets transmitted by different nodes collide with each other. In this case, other cooperative nodes sense the collisions and give up transmitting their own HTS packets. When the sender senses the collisions, it transmits a negative RTS (NRTS) packet, which includes the addresses of the nodes whose HTS packets have been received correctly. After the nodes that lead to the HTS packet collisions receive the NRTS packet, they realize that their HTS packets are not received correctly. On receiving the NRTS packet, these nodes select a random time from (0, *T*_R_) as the new access delay of the HTS packets and begin counting. Meanwhile, these cooperative nodes keep sensing the channel. When a cooperative node senses the HTS packet transmission from another cooperative node, it pauses the counting and continues the counting after the current HTS transmission. It transmits its HTS packet at the end of its access delay counting. If the waiting time exceeds *T*_R_, the cooperative contention phase ends because there will be no more HTS packet transmissions. The sender calculates the optimal transmission powers based on the received HTS packets and declares the results through an OPD packet. The reason for this HTS retransmission strategy is that the nodes with higher cooperative gain should always have priority to be employed than the nodes with lower cooperative gain.

On receiving the OPD packet, each cooperative node knows whether it will take part in the following data packet transmission phase and the value of its corresponding transmission power. Then the PO-CMAC protocol enters into the data packet transmission phase.

Combining this cooperative node selection strategy with the LETPO algorithm, we can consider both the average energy consumption of each data packet transmission (i.e., the nodes’ energy efficiency) and the energy utilization of the network, thus extending the network lifetime efficiently.

### 5.3. Data Packet Transmission Phase

**Figure 6:**
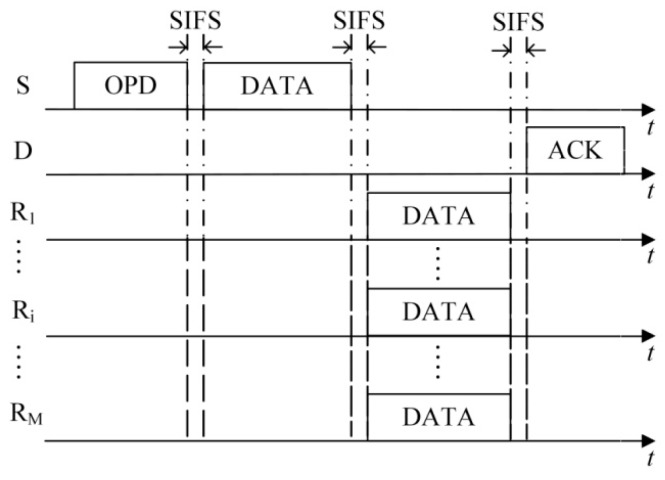
Data packet transmission phase.

In the data packet transmission phase, the data packets are transmitted from the sender to its recipient through two-hop manner, i.e., from the sender to the cooperative nodes and then from the cooperative nodes to the recipient. As shown in Figure 6, at the beginning of the data packet transmission phase, the sender broadcasts its data packet to its cooperative nodes and recipient using the optimal transmission power *P*_S_ calculated during the cooperative node selection phase. Since the optimal transmission power *P*_S_ is lower than *P*_D_, which is a threshold of correctly decoding in direct transmission, the recipient cannot decode the data packet except receiving and saving it. The cooperative nodes forward the data packet to the recipient using the optimal transmission powers declared in the OPD packet. Then the recipient decodes the data packet based on the data packet sent from the sender and those forwarded from the cooperative nodes by using MRC technique. If the recipient successfully decodes the data packet, it will reply with an acknowledgment (ACK) packet to the sender to confirm its successful data packet reception.

If the recipient cannot decode the data packet successfully, the data packet needs to be retransmitted. Because the recipient has saved the data packets from the sender and the cooperative nodes, it only needs an extra data packet transmission in a certain power in order to correctly decode the packet without wasting too much energy. In the cooperative node selection phase, the cooperative nodes transmit their HTS packets to the sender in an order of their cooperative ability to participate in the cooperative transmission. Therefore, we should choose the cooperative node with the highest cooperative gain to retransmit the data packet.

**Figure 7:**
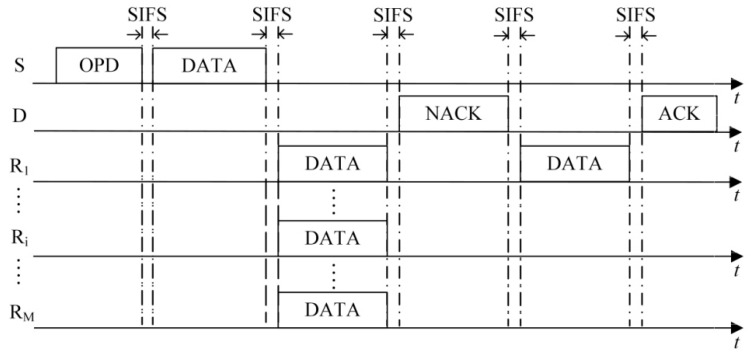
Successful data packet transmission after one-time retransmission.

**Figure 8:**
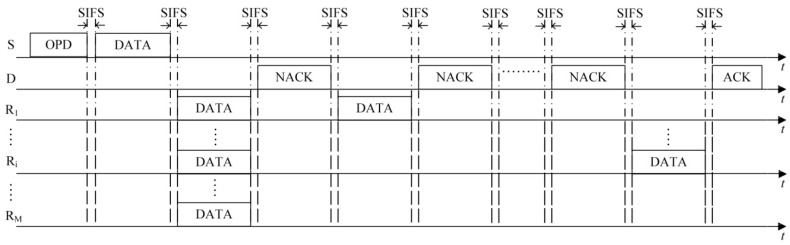
Successful data packet transmission after multiple retransmissions.

As shown in Figure 7, if a data packet is not correctly decoded, a negative ACK (NACK) packet is transmitted by the recipient. On receiving this packet, all nodes that participate in the current data packet transmission record that this is the first NACK packet transmitted by the recipient. Then, the first cooperative node to transmit its HTS packet during the cooperative node selection phase retransmits the data packet. Furthermore, the recipient decodes the data packet combined with the previously-received data packets by MRC technique. If succeeded, the recipient replies with an ACK packet. Otherwise, as shown in Figure 8, the recipient continues to transmit a NACK packet, and the second cooperative node to transmit its HTS packet during the cooperative node selection phase retransmits the data packet. This retransmission process repeats until the data packet is successfully decoded. If all selected cooperative nodes have retransmitted the data packet and the recipient still fails to decode the packet, the current data packet transmission fails and the sender will directly transmit the data packet to its recipient using the transmission power *P*_D_.

## 6. Simulation Results

In this part, we employ C programming language for simulation and give performance comparison among the lifetime-extended PO-CMAC protocol, direct transmission (DT) protocol and energy efficiency cooperative relay (EE-CR) protocol proposed in [34]. To measure the effect of different protocols on the energy efficiency and network lifetime, we investigate the network lifetime, the average number of data packets transmitted by each node during the network lifetime, network energy utilization and throughput. Herein, the network lifetime is defined as the duration time from the network initialization to the time when the first node exhausted its energy [36,43]. The network energy utilization is defined as the ratio of the energy used during the network lifetime to the initial network energy. High network energy utilization indicates the energy of the network is fully used. The throughput is defined as the ratio of the time on the channel to be used for successful data packet transmissions by all the nodes in the network to the total elapsed time.

### 6.1. Simulation Environment

All nodes in the network are randomly distributed in a rectangle area of 100 m × 100 m. Each node generates data packets with a fixed length according to a Poisson distribution with the arrival rate *λ*. The default value of *λ* is 1 packet/s. The recipient of each data packet is randomly selected from the neighbor nodes of the sender. Each node has initial energy of 1 J. The wireless channel is a Rayleigh fading channel and the path loss exponent is 3. All protocols operate in a single rate mode. In order to guarantee that the transmission rates from the sender to its recipient are the same in different protocols, if the direct transmission is used, the bandwidth utilization is *R*, and if the cooperative transmission is used, the bandwidth utilization is 2*R*. The default value of *R* is 2 bit/s/Hz. In the simulation, the energy consumption of each data packet transmission includes not only the energy to transmit the data packet but also the energy to transmit the corresponding control packets. Let *L*_PHY_, *L*_MAC_, *L*_CRTS_, *L*_NRTS_, *L*_CCTS_, *L*_HTS_, *L*_ACK_, *L*_NACK_, *L*_OPD_ and *L*_DATA_ be the lengths of PHY header, MAC header, CRTS, NRTS, CCTS, HTS, ACK, NACK, OPD and data packets, respectively. Table 1 gives the default parameter settings used in the simulation.

In WSNs, the traffic load distribution is an important influence factor on network lifetime. Therefore, we investigate the network performance at both uniform traffic load and nonuniform traffic load.

### 6.2. Network Performance at Uniform Traffic Load

**Figure 9:**
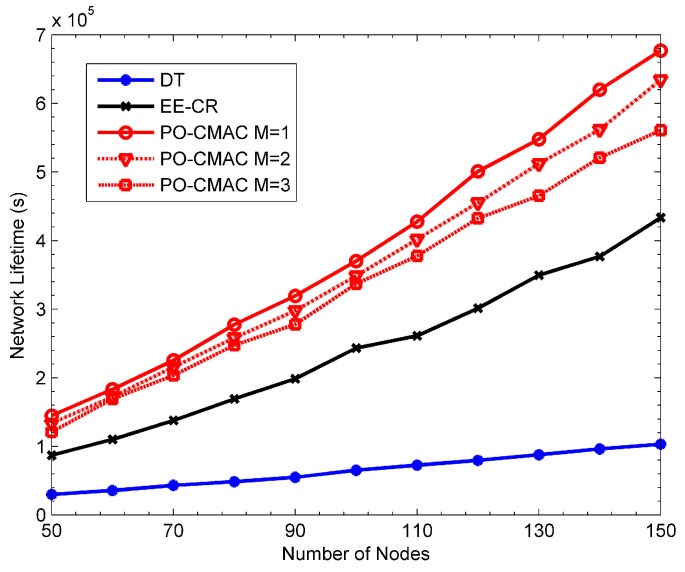
Network lifetime with varying *N* at uniform traffic load.

**Figure 10:**
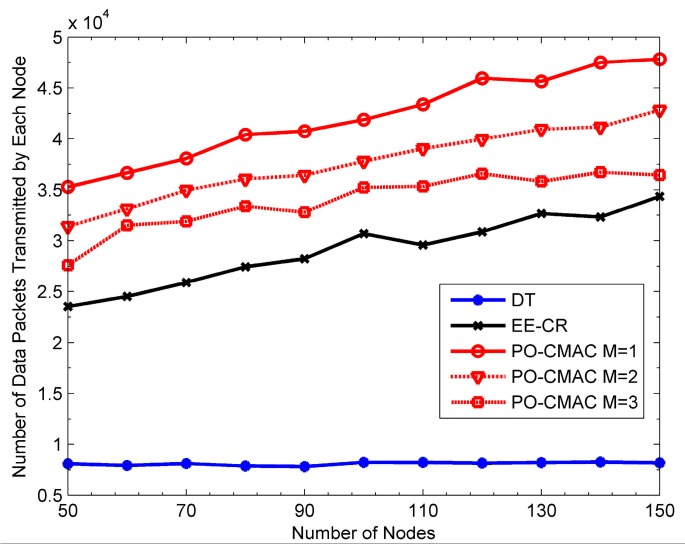
Number of data packets transmitted by each node with varying *N* at uniform traffic load.

**Figure 11:**
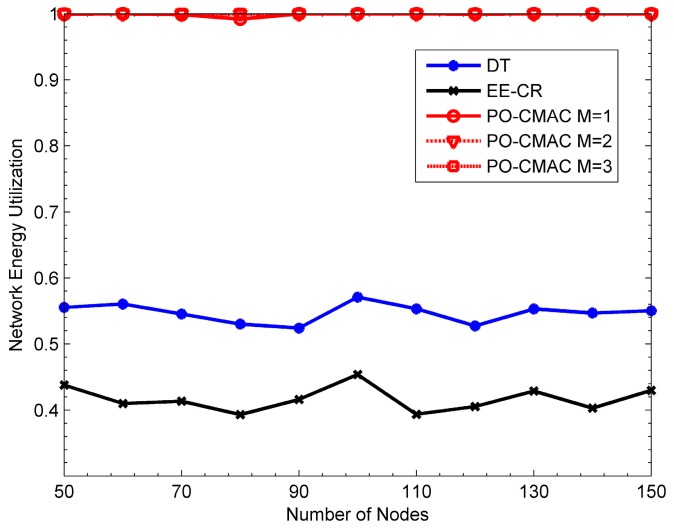
Network energy utilization with varying *N* at uniform traffic load.

Figure 9, Figure 10 and Figure 11 show the performance of the DT, EE-CR and PO-CMAC protocols at uniform traffic load, respectively. Herein, the number of nodes *N* in the network changes from 50 to 150. As shown in Figure 9 and Figure 10, the PO-CMAC protocol outperforms the DT and EE-CR protocols in both network lifetime and the number of data packets transmitted by each node. Furthermore, in the case of uniform traffic load, the PO-CMAC protocol with *M* = 1 has the longest network lifetime and the most data packets transmitted by each node. When *N* = 150 and *M* = 1, the network lifetime and the number of data packets transmitted by each node of the PO-CMAC protocol are 56.17% and 39.24% higher than those of the EE-CR protocol, respectively. The advantage of the PO-CMAC protocol mainly originates from the following two aspects.

(1) The cooperative transmission obtains spatial diversity gain by considering transmission link quality and transforming the direct transmission link from the sender to its recipient to the two-hop cooperative transmission links, i.e., from the sender to the cooperative nodes and from the cooperative nodes to the recipient. If the direct transmission link between the sender and its recipient is in deep fading, in traditional direct transmission, the sender can only increase the transmission power to guarantee the transmission reliability. However, in the cooperative transmission, considering the independent fading channel, the sender can select the cooperative nodes with the best cooperative transmission link to cooperate. Therefore, it can obtain diversity gain and guarantee the transmission reliability with low transmission power. As shown in Equations (22) and (23), in the cooperative node selection phase of the PO-CMAC protocol, the cooperative nodes with higher channel gain is chosen with higher priority, which ensures that the cooperative transmission fully explores the spatial diversity gain obtained by node cooperation and consumes less energy than the direct transmission.
(2) Meanwhile, the PO-CMAC protocol considers the impact of the residual energy balance of the local network on the network lifetime. By balancing the energy consumption of nodes in local network, it avoids decreasing network lifetime due to the early energy exhaustion of some nodes. Usually when the cooperative transmission achieves spatial diversity, it also leads to extra energy consumption of some nodes due to their cooperative transmissions for other nodes, which aggravates the non-uniform energy consumption of the nodes in local network. However, in the PO-CMAC protocol, the LETPO algorithm makes the nodes with little residual energy consume energy as less as possible in the current data packet transmission by maximizing the minimum residual energy of each associated node, thus avoiding part of nodes consuming their energy too soon and balancing the energy consumption of the local network. Therefore, the PO-CMAC protocol outperforms the EE-CR protocol, which also adopts cooperative transmission manner.

The results in Figure 11 also validate the above conclusions. The network energy utilization of the EE-CR protocol is between 0.4 and 0.45, which is even lower than that of the DT protocol (i.e., from 0.5 to 0.6). The reason for this phenomenon is that the cooperative transmission employed in the EE-CR protocol leads to extra energy consumption of cooperative nodes besides their own data packet transmissions, which intensifies the uneven energy consumption of each node in the network and leads to the early energy exhaustion of some nodes. Namely, its network energy is not fully utilized. The network energy utilization of the PO-CMAC protocol is close to 1. That is to say, the residual energy of the network is very little and its network energy is fully used. This is also one of main reasons that the PO-CMAC protocol performs better than the DT and EE-CR protocols in terms of network lifetime and the number of data packets transmitted by each node.

From Figure 9 and Figure 10, when *M* = 1, the network lifetime is the longest and the number of data packets transmitted by each node is the largest. Furthermore, as *M* increases, both of them decrease. This is because when the network traffic load is uniform, different nodes have the same arrival rates and then consume the energy in a similar way. In this case, it only needs to select one cooperative node with the best channel quality in the cooperative node selection phase and then balances the residual energy of the sender and this cooperative node through the LETPO algorithm. By this mechanism, both high energy utilization of the network and high energy efficiency of the nodes are guaranteed, and thus the network lifetime is further extended. Figure 11 also shows this point. When *M* = 1, the energy utilization of the PO-CMAC protocol is nearly 1 and there is no more room for performance improvement. Moreover, when *M* = 1, the overhead is the least and the energy efficiency is the highest. Therefore, the network lifetime is the longest.

### 6.3. Network Performance at Nonuniform Traffic Load

**Figure 12:**
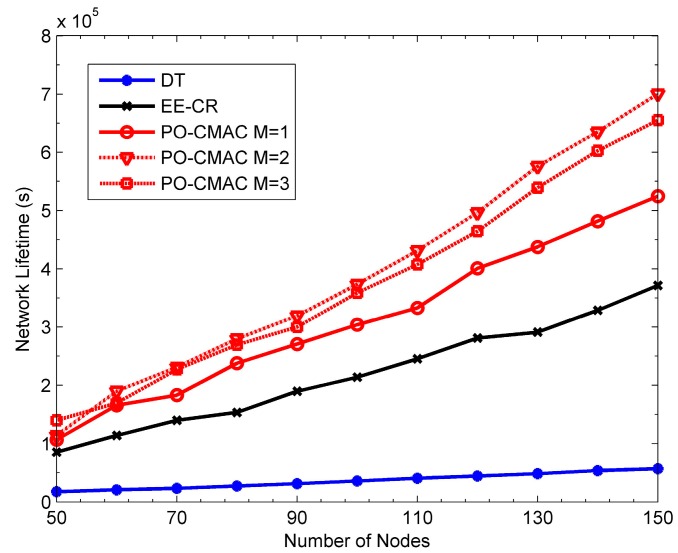
Network lifetime with varying *N* at nonuniform traffic load.

**Figure 13:**
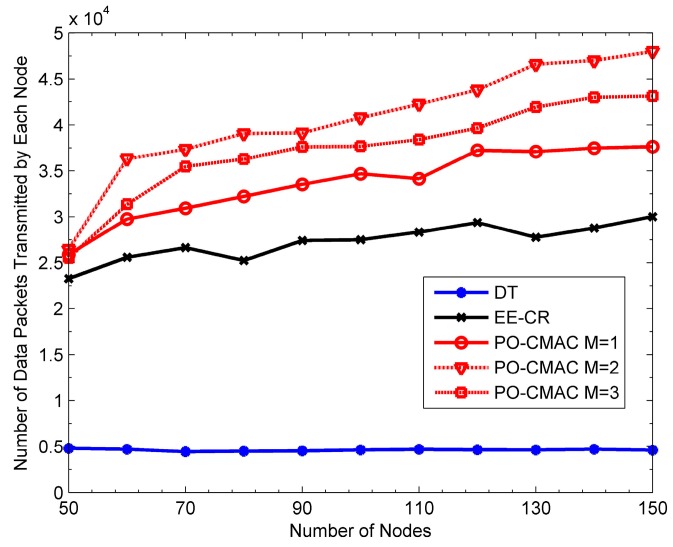
Number of data packets transmitted by each node with varying *N* at non-uniform traffic load.

**Figure 14:**
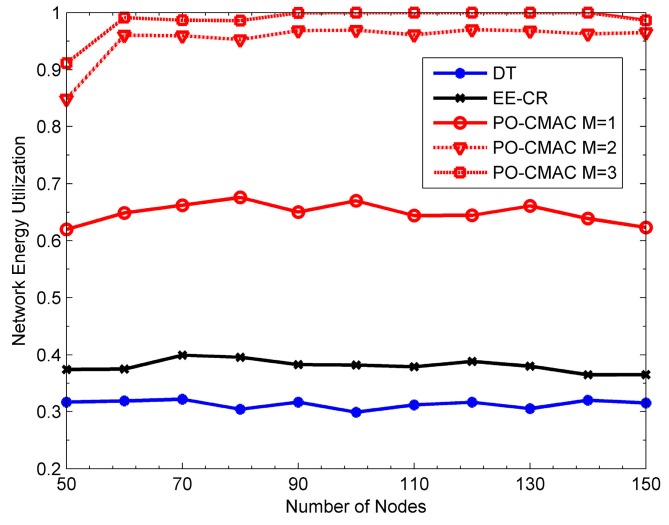
Network energy utilization with varying *N* at non-uniform traffic load.

Figure 12, Figure 13 and Figure 14 show the network lifetime, the number of data packets transmitted by each node and network energy utilization of the DT, EE-CR and PO-CMAC protocols at non-uniform traffic load, respectively. Herein, the packet arrival rates of half nodes are 1.5 packets/s and those of the other half are 0.5 packets/s. From the figures, at non-uniform traffic load, the PO-CMAC protocol still outperforms the DT and EE-CR protocols. When *N* = 150 and *M* = 1, the network lifetime and the number of packets transmitted by each node of the PO-CMAC protocol are 41.11% and 25.40% higher than those of the EE-CR protocol, respectively. Furthermore, when *N* = 150 and *M = 2*, they are 88.53% and 59.98% higher than those of the EE-CR protocol, respectively. This can be explained as follows. The non-uniform traffic load intensifies the energy consumption difference of different nodes and results in that the nodes with higher packet arrival rate exhausted their energy too early due to more data packet transmissions, thus reducing the network energy utilization and decreasing the network lifetime. However, the PO-CMAC protocol not only improves node energy efficiency through diversity gain obtained by node cooperation but also balances the local network energy consumption through the LETPO algorithm. Any nodes with low packet arrival rate can use more energy to cooperate the nodes with high packet arrival rate or other nodes. Therefore, the PO-CMAC protocol can reduce the difference of energy consumption caused by different packet arrival rates, make full use of the network energy and extend the network lifetime.

In addition, there are still some differences between the performance at uniform network traffic load and that at non-uniform network traffic load. Compared to the results at uniform network traffic load in Figure 9, Figure 10 and Figure 11, the DT and EE-CR protocols have less performance at non-uniform traffic load. This is because different packet arrival rates of different nodes lead to the difference in energy consumption, and some nodes will exhaust their energy too fast due to too many data packet transmissions. The performance of the PO-CMAC protocol with *M* = 1 at non-uniform traffic load is also lower than that at uniform traffic load. According to Equations (22) and (23), when *M* = 1, the PO-CMAC protocol chooses only one cooperative node with the best link quality in cooperative node selection phase, and then the sender and the cooperative node transmit data packets in the optimal transmission powers calculated by the LETPO algorithm. However, at non-uniform traffic load, the residual energy of nodes differs greatly from each other. The selected cooperative node with the highest channel gain in cooperative node selection phase probably has consumed too much energy due to too many data packet transmissions. In this case, if the node still takes part in the cooperative transmission, it will speed up its energy consumption and will not balance the energy consumption of the local network, thus reducing the network lifetime. As seen from Figure 14, the energy utilization of the PO-CMAC protocol with *M* = 1 drops to between 0.6 and 0.7.

As shown in Figure 12 and Figure 13, the PO-CMAC protocol with *M* = 1, 2 and 3 outperforms other protocols because the performance advantage of the PO-CMAC protocol is derived not only from the diversity gain obtained by cooperative transmissions, but also from the effect of the LETPO algorithm on balancing the local network energy consumption. The LETPO algorithm balances the energy consumption of different senders and cooperative nodes in the process of each data packet transmission, which finally balances the energy consumption of the whole network. For *M* = 1, the LETPO algorithm only balances the residual energy of the sender and a single cooperative node. Therefore, from the global viewpoint, the effect of network energy balance cannot compensate the residual energy differences caused by non-uniform traffic transmissions. For *M* = 2, the LETPO algorithm can balance the residual energy of the sender and two cooperative nodes during each data packet transmission, thereby accelerating the effect of balancing the whole network energy consumption. More specifically, it is more likely to select the cooperative nodes with both good channel quality and sufficient residual energy for cooperation transmissions, which takes full advantage of both diversity gain and the cooperative nodes with more residual energy. Therefore, it resolves the unbalance problem of energy consumption due to non-uniform traffic transmissions and extends the network lifetime efficiently. As shown in Figure 14, when *M* = 2, the energy utilization of the PO-CMAC protocol reaches 0.95. If *M* is further increased, there is no room for improving energy utilization, and instead, there will be more energy overhead for each data packet transmission. Therefore, when *M* = 3, although the energy utilization of the PO-CMAC protocol nearly reaches 1, its network lifetime and average number of data packets transmitted by each node become lower than those when *M* = 2.

From the above results, it can be seen that the PO-CMAC protocol outperforms the DT and EE-CR protocols in terms of the network lifetime, the average number of data packets transmitted by each node and energy utilization at both uniform and non-uniform traffic load. In the PO-CMAC protocol, the parameter *M* can be changed according to the traffic load to achieve more satisfactory performance. Generally, the PO-CMAC protocol with *M* = 1 is the best in the presence of uniform traffic load, and the PO-CMAC protocol with *M* = 2 is suitable for non-uniform traffic load.

### 6.4. Impact of M on Protocol Performance

**Figure 15:**
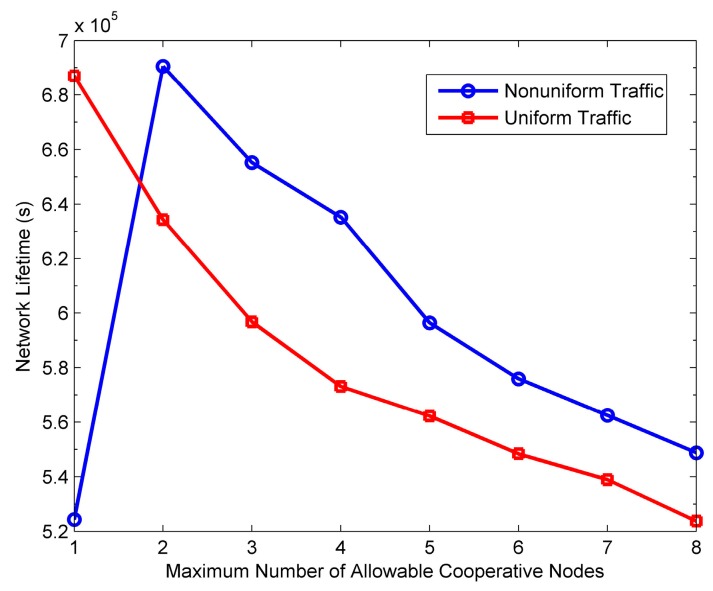
Network lifetime vs. *M*.

**Figure 16:**
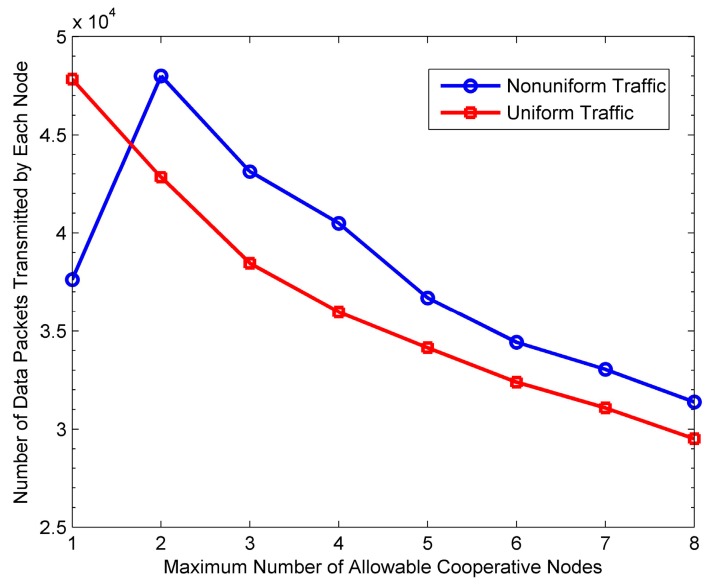
Number of data packets transmitted by each node vs. *M*.

**Figure 17:**
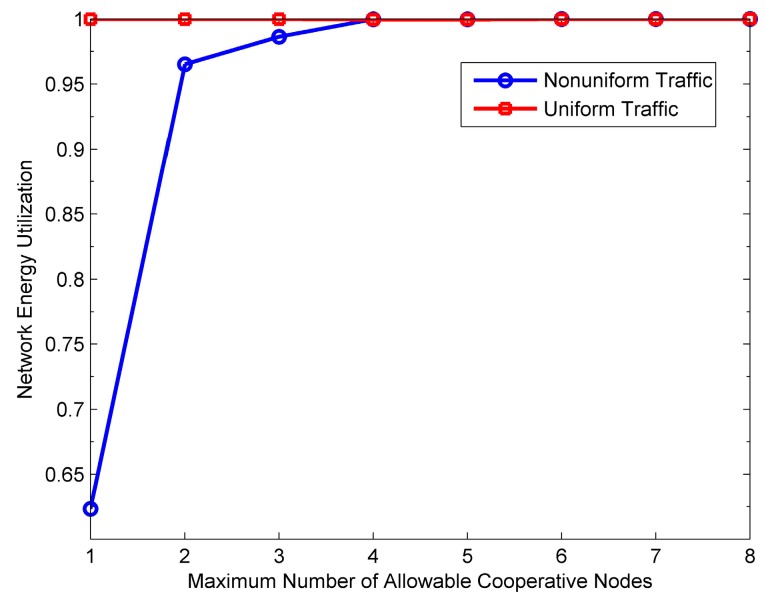
Network energy utilization vs. *M*.

**Figure 18:**
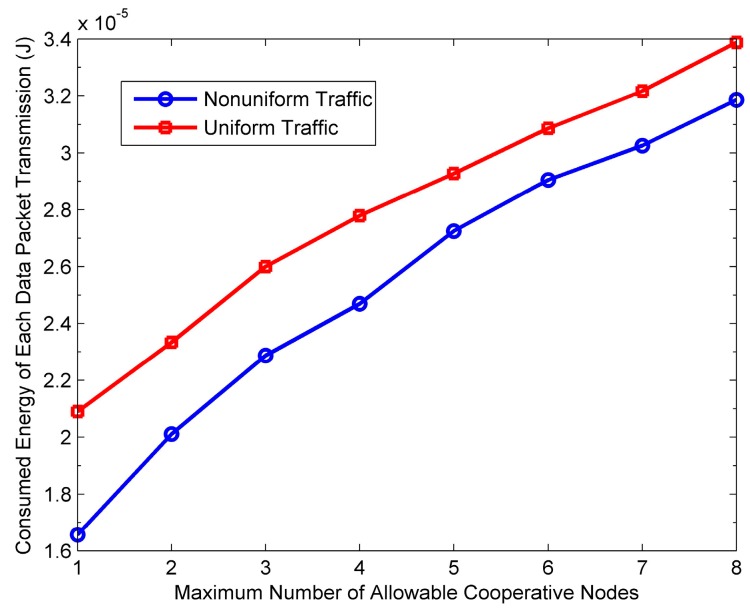
Average consumed energy of each data packet transmission vs. *M*.

Figure 15, Figure 16, Figure 17 and Figure 18 show the impact of *M* on the performance of the PO-CMAC protocol. Herein, the number of nodes *N* is 150. As shown in Figure 15 and Figure 16, when the network traffic load is uniform, the network lifetime and the average number of data packets transmitted by each node reach the maximum values at *M* = 1, and then decreases as *M* increases. Furthermore, at non-uniform network traffic load, they increase at first and then decrease. All their maximum values are obtained at *M* = 2. This is the tradeoff results between the less energy consumption as *M* decreases and the better effect on balancing network energy consumption as *M* increases. According to Equation (17), with a certain initial energy, the total number of data packet transmissions of the network is proportional to the network energy utilization and inversely proportional to the average energy consumption of each data packet transmission. For this reason, Figure 17 and Figure 18 reflect this tradeoff relationship with the variation of *M*.

As shown in Figure 17, at the uniform network traffic load, the energy utilization of the PO-CMAC protocol is always close to 1. This is because each node can transmit almost the same number of data packets and consume similar amount of energy, which leads to similar residual energy. In this case, selecting a single cooperative node with the best channel quality (i.e., *M* = 1) can obtain cooperative diversity gain, and meanwhile the LETPO algorithm can balance the network energy consumption efficiently, which guarantees high energy utilization. At this time, if *M* is further increased, there is no room for improving energy utilization. At the non-uniform network traffic load, the network energy utilization increases significantly with the increase of *M* and then approach to 1 smoothly. This is because the different packet arrival rates lead to the differences in the energy consumption and residual energy of each node. For *M* = 1, the LETPO algorithm can only balance the residual energy of the sender and a single cooperative node. Furthermore, it is possible that both these two nodes have little residual energy. Therefore, it might fail to balance the local network energy consumption when the network traffic load is non-uniform, which leads to low network energy utilization. With the increase of *M*, it is more possible for the PO-CMAC protocol to choose cooperative nodes with both higher quality link and sufficient residual energy in cooperative node selection phase. Meanwhile, the LETPO algorithm can balance the residual energy of nodes within a larger local network, thus improving the network energy utilization. Figure 17 shows that, when *M* is changed from 1 to 2, the improvement of the energy utilization is the largest.

Furthermore, as shown in Figure 18, the average energy consumption of each data packet transmission increases with the increase of *M* at both uniform traffic load and non-uniform traffic load. There are two reasons for this phenomenon. Firstly, the increase of *M* leads to more HTS packet transmissions in cooperative node selection phase, which results in extra energy consumption overhead. Secondly, as *M* increases, it is more likely for the sender to choose the cooperative nodes with poor channel quality but sufficient residual energy to take part in cooperative transmission, which also increases the energy consumption of each data packet transmission.

Considering both Figure 17 and Figure 18, on one hand, the increase of *M* is better for balancing the residual energy of the local network and improving network energy utilization. On the other hand, it increases the average energy consumption of each data packet transmission, thus reducing the energy efficiency. Therefore, according to Equation (17), in different cases, in order to transmit more data packets and extend network lifetime, we should select the optimal value of *M* to guarantee high energy utilization and avoid low energy efficiency.

Moreover, in Figure 16, when *M* is large, the average number of data packets transmitted by each node at non-uniform traffic load is a little higher than that at uniform traffic load. The reason is that, when the packet arrival rates of nodes in the network are different, their residual energy is different from each other and the nodes with more residual energy are more likely to take part in cooperative transmissions. Namely, more energy is used for cooperative transmissions and thus more cooperative gain is obtained, which leads to less energy consumption of each data packet transmission. The results in Figure 18 validate the above viewpoint.

**Figure 19:**
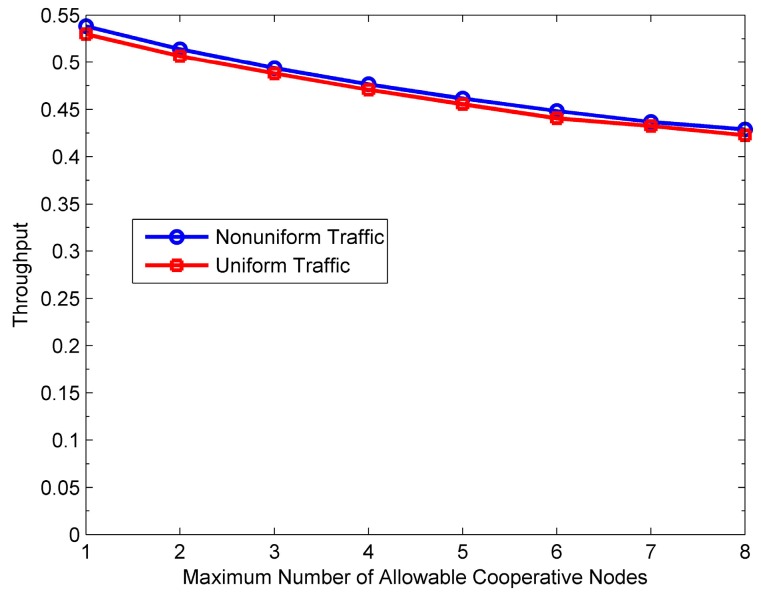
Throughput vs. *M*.

Figure 19 shows the network throughput with varying *M*. It can be seen that in any case, the throughput decreases with the increase of *M* because more HTS packet transmissions lead to more transmission delay, thus reducing the throughput to some extent. Therefore, comprehensively considering Figure 15, Figure 16 and Figure 19, if the network lifetime and the number of data packets transmitted by each node already achieve the maximum, it should not increase *M* anymore to avoid reducing the throughput.

### 6.5. Throughput

From the above simulation results, the PO-CMAC protocol has a significant improvement on energy performance. However, in order to coordinate the contention and access of cooperative nodes, it introduces extra overhead, which unavoidably increases the transmission delay and reduces the network throughput. To resolve this decrease in throughput, for a high throughput requirement of the network, we can improve the data transmission rate between the sender and the recipient in the PO-CMAC protocol (i.e., improve the bandwidth utilization in the same bandwidth). The increase of the data transmission rate will increase energy consumption of each data packet transmission, thus leading to the reduction in network lifetime. However, compared to the DT protocol with a low data transmission rate, the PO-CMAC protocol still extends the network lifetime efficiently in this case. To illustrate this case, we give the results of the DT protocol with *R* = 2 bit/s/Hz, and the PO-CMAC protocol with *R* = 2 and 4 bit/s/Hz, respectively.

**Figure 20:**
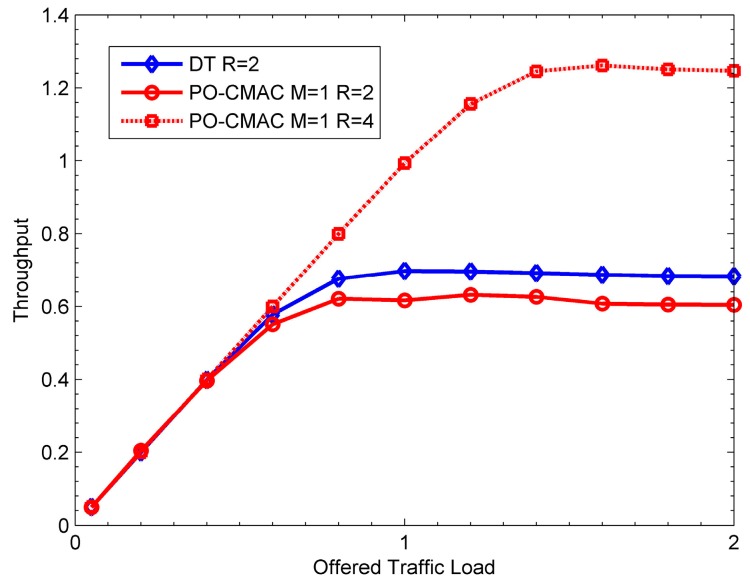
Throughput vs. offered traffic load.

Figure 20 shows the network throughput with the variation of offered traffic load. When *R* = 2 bit/s/Hz, the throughput of the PO-CMAC protocol is 0.6 and lower than that of the DT protocol (i.e., 0.68) due to extra control packet transmissions. However, when *R* = 4 bit/s/Hz, its throughput is 1.25, which is distinctly higher than that of the DT protocol.

**Figure 21:**
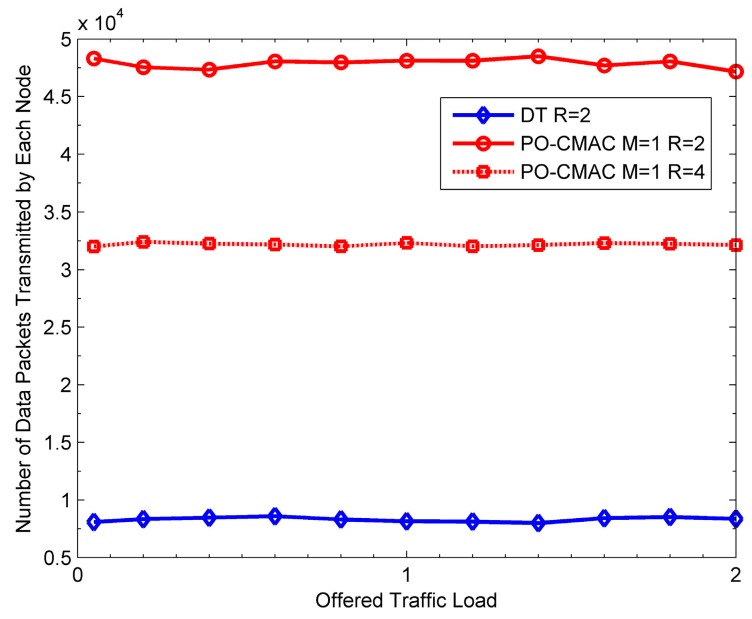
Number of data packets transmitted by each node vs. offered traffic load.

Figure 21 shows the corresponding energy efficiency performance. Because the network lifetime will contain a certain idle time when the network is unsaturated, we use the average number of data packets transmitted by each node to reflect the energy efficiency performance and network lifetime of each protocol. From the figure, when *R* = 2 bit/s/Hz, the PO-CMAC protocol has much higher average number of data packets transmitted by each node than the DT protocol. When *R* = 4 bit/s/Hz, the throughput of the PO-CMAC protocol increases, but its average energy consumption of each data packet transmission also increases, which leads to a decrease of the average number of data packets transmitted by each node. However, in this case, it is still higher than that of the DT protocol with *R* = 2 bit/s/Hz.

Figure 20 and Figure 21 also illustrate two conclusions. Firstly, when the data transmission rates between the sender and its recipient are the same, the throughput of the PO-CMAC protocol slightly decreases, but its network lifetime is significantly extended. Secondly, for a high requirement in terms of throughput, we can improve the equivalent data transmission rate between the sender and its recipient (i.e., improve the transmission powers of the sender and its cooperative nodes to achieve the expected bandwidth utilization). In this case, although the network lifetime of the PO-CMAC protocol decreases, it is still higher than that of the DT protocol, and its throughput is also much higher than that of the DT protocol. Namely, besides the better performance on network lifetime, the PO-CMAC protocol proposed in this paper can also balance the performance between the throughput and energy efficiency to achieve a significant overall advantage compared to the DT protocol.

## 7. Conclusions

In this paper, we proposed a network lifetime-extended power-optimized cooperative MAC protocol for WSNs. In the protocol, the LETPO algorithm is used to calculate optimal transmission powers of the sender and its cooperative nodes under certain constraint conditions based on linear programming and greedy algorithm, which obtains diversity gain and transfers part of energy consumption of one data packet transmission from the sender to the cooperative nodes, thus balancing the local network energy consumption and extending the network lifetime. Furthermore, a cooperative node selection scheme is used to efficiently choose cooperative nodes with high channel gain and avoid HTS transmission collisions by limiting the maximum number of cooperative nodes to transmit HTS packets and coordinating their access delay. Simulation results show that compared to the DT and EE-CR protocols, the proposed PO-CMAC protocol can efficiently improve the network lifetime, average number of data packets transmitted by each node and network energy utilization at both uniform and non-uniform network traffic load. Furthermore, it can adapt to different network traffic load by adjusting the parameter *M*, thus extending the network lifetime efficiently. In general, the PO-CMAC protocol with *M* = 1 is the best for uniform traffic load, and the PO-CMAC protocol with *M* = 2 is suitable for non-uniform traffic load.

In the future work, we will improve our protocol by optimizing both throughput and energy consumption performance in a multiple data rate network. We also plan to embed a duty cycling mechanism into our protocol to further extend network lifetime and improve throughput. 

## Figures and Tables

**Table 1:** Parameter settings.

Parameter	Value	Parameter	Value
*B*	10 kHz	*P*_max_	50 mW
*N*_0_	−80 dBm	*T*_W_	100 μs
*T*_R_	50 μs	*L*_MAC_	272 bits
*L*_PHY_	192 bits	*L*_CRTS_/*L*_NRTS_	160 bits
*L*_CCTS_/*L*_HTS_/*L*_ACK_/*L*_NACK_	112 bits	*L*_OPD_	160 bits
*L*_DATA_	1000 bits	SIFS	10 µs
